# Supplementary figures and images for: Large-scale differences in diversity and functional adaptations of prokaryotic communities from conserved and anthropogenically impacted mangrove sediments in a tropical estuary
Source: PeerJ. 2021 Sep 23;9:e12229. doi: 10.7717/peerj.12229 (PMC8465992; doi:10.7717/peerj.12229)

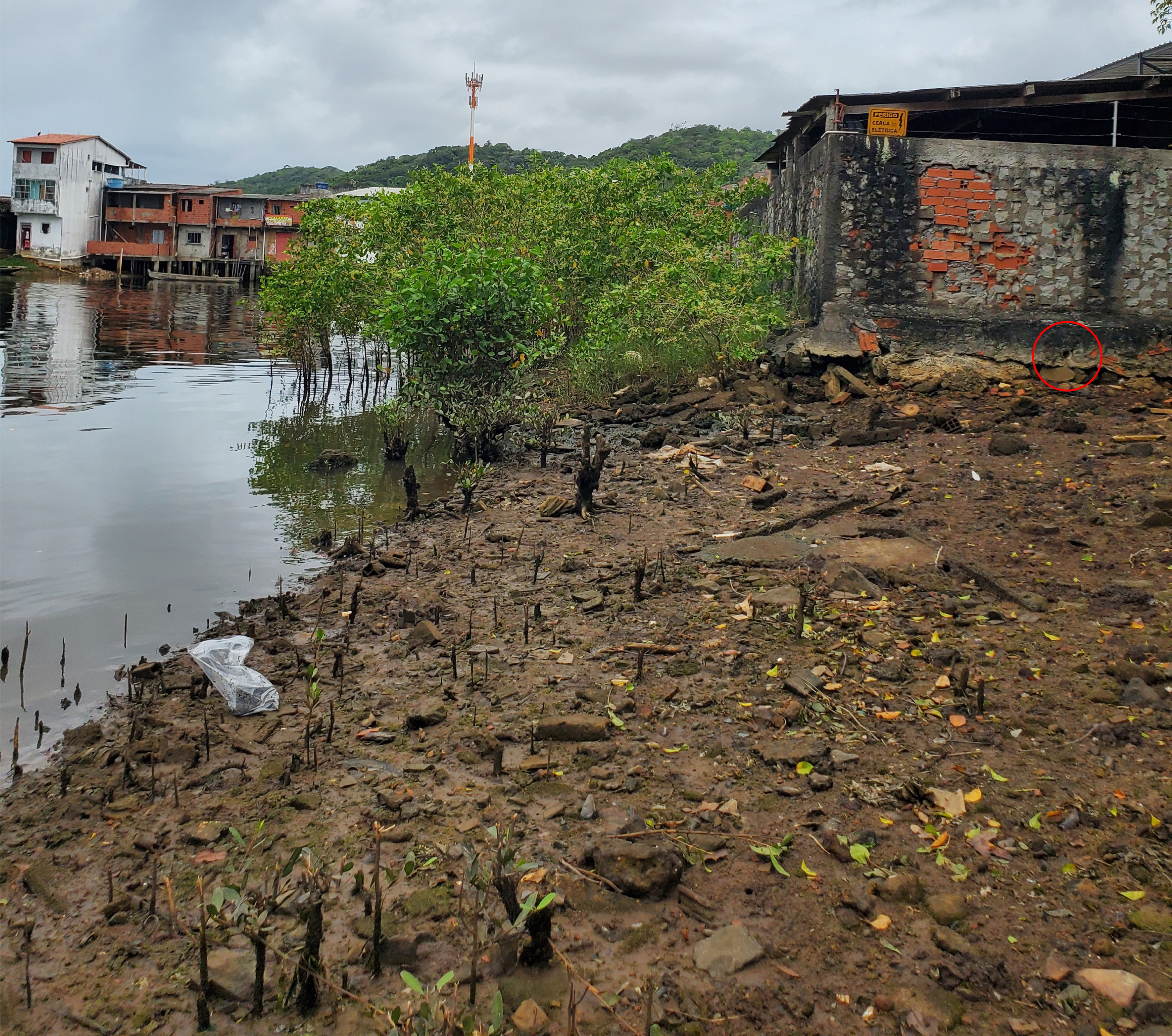

Supplement: Supplemental Information 1 — Figure shows clear signs of human disturbances in the mangrove. We emphasize the pipe coming from inside the construction, discharging domestic sewage directly in the mangrove sediments. [file peerj-09-12229-s001.jpg]
