# Supplementary material for: Large-scale differences in diversity and functional adaptations of prokaryotic communities from conserved and anthropogenically impacted mangrove sediments in a tropical estuary
Source: PeerJ. 2021 Sep 23;9:e12229. doi: 10.7717/peerj.12229 (PMC8465992; doi:10.7717/peerj.12229)
Supplement: Supplemental Information 2 [file peerj-09-12229-s002.pdf]

Supplemental Methods section:

Read Trimming

Trimmomatic (Bolger, Lohse and Usadel 2014) was used to filter and trim demultiplexed sequences (ILLUMINACLIP:TruSeq3-PE.fa:2:30:10 LEADING:3 TRAILING:3 SLIDINGWINDOW:4:15 MINLEN:100). A minimum average read quality score of 15 was required for inclusion while the sliding window cuts any read at the point where the median quality score over a 4 nucleotide window is less than 15.

Read-pairing and de-noising

For conserved site reads we used a tandem pairing process as QIIME2 uses an implementation of vsearch for read pair joining (‘vsearch join-pairs’) which has a lower criteria of a minimum of 10 base-pairs of overlap. To extend vsearch with accurate read-pair joining below this cut-off we used a custom script (jantar.py) that would attempt to combine read-pairs that had failed to combined with vsearch. In order to be combined a read pair must have a “perfect” (starting from tails of reads, with no mismatches, in the correct orientation) 7bp overlap or 10bp with a single mismatch. Reads were denoised using ‘dada2 denoise-single’, default settings using ‘--p-trim-left 3 and --p-trunc-len 0’.

DADA2 as implemented by QIIME2 was used for read-pairing and denoising of the Impacted site data.

QIIME2

Phylogeny was determined using ‘phylogeny align-to-tree-mafft-fasttree’ with default settings. Minimum (48638) and maximum (106724) read depths were determined using ‘table_dada2.qzv’. Alpha-rarefaction was then calculated using the rooted MAFFT tree and the maximum read depth.

Alpha and Beta diversities were calculated using ‘diversity core-metrics-phylogenetic’ using the minimum read depth (‘--p-sampling-depth’) and the rooted MAFFT tree. Alpha-diversity was evaluated using the Kruskal-Wallis test, Beta-diversity evaluated using PERMANOVA. Rarefied data did not violate Kruskal-Wallis homoscedasticity assumption using Levene’s test, and betadisper as implemented in the vegan R package was used to test for PERMANNOVA’s multivariate homoscedasticity assumption.

Taxonomy was calculated using ‘feature-classifier classify-sklearn’ and plotted using ‘taxa barplot’.

Finally, gneiss was used to calculate the hierarchical correlation-clustering of unnormalized taxa counts constrained to the levels of Family and greater using ‘gneiss correlation-clustering’. A pseudo-count of 1 was used for handling log-transforms. This was plotted using ‘dendrogram-heatmap’ for the meta-data column ‘Site’.

PICRUSt2

PICRUSt2 was run twice using '--stratified' and '--per_sequence_contrib' options. One run was set to limit NSTI thresholds to 0.15, while the other (and data set subsequently used) was ran with the default NSTI threshold of 2.0.

Taxonomic abundance analysis

Novembro.py normalizes the ASV read abundances generated by QIIME2 into their corresponding taxa. This requires feature-table.tsv and taxonomy.tsv files. While the taxonomy.tsv file can be found by unzipping the taxonomy.qza file the feature-table.tsv needs to be generated using a biom conversion command (eg. 'biom convert -i feature-table.biom -o feature-table.biom.txt --to-tsv')

Novembro.py iterates from the lowest to the highest taxonomic level combining ASV read abundances into their corresponding taxa. Replicate normalizations are downsampled to match the lowest ASV abundant replicate. We use novembro.py to calculate zone specific taxonomic enrichment. This is accomplished by first using chi2 to compare the replicates of each zone against the others. To be deemed significant a zone must have an greater than a 5% effect size and p-value less than, or equal to, 0.05 relative to both other sites.

Taxa that are found to be significantly enriched are saved to a tab-delimited file along with their log10 transformed abundances.

Zone specific differential KO abundances

First, we generate Monte Carlo samples of each KO distribution of the unstratified metagenome predictions (eg. 'pred_metagenome_unstrat.tsv') using ALDEx2's centered log-transformed ('aldex.clr') module. We then use ALDEx2's GLM ANOVA test ('aldex.kw'), taking those KOs with an expected p-value ('glm.ep') of less than, or equal to, 0.05 to be significant.

Potential functional abundance analysis

Sigilo.py performs several functions to aid in the visualization and analysis of PICRUSt2 and ALDEx2 generated KO data.

Generate heatmap: KO enrichment heatmaps can be generated using '--generate_heatmap', this function combines the predicted functional abundances of KOs identified by ALDEx2 as significant into their corresponding KEGG Ortholog pathways, performs a log10 transform and plots them as heatmaps.

Correlate ASV with Functional Abundance: Using the '--asv2fa' command we first combine all ASVs into their corresponding taxa at a given level (for Family, level = 4) and then calculate the total functional abundance for those combined ASVs to derive the predicted functional abundance of a given taxa.

Correlate ASV with NSTI: Using the '--asv2nsti' command we first combine all ASVs into their corresponding taxa at a given level (for Family, level = 4) and then combine the associated NSTI values for those ASVs for each taxa. We can then use these to identify the mean, median, and standard deviation of NSTI scores for each given taxa.

Supplemental data section

Variable	C1	C2	C3	I1	I2	I3	I/C	H	p-value	
Temperature (C)	25.00	29.00	29.00	29.70	29.30	29.00	1.06	2.63441	0.10457	
Dissolved Oxygen (mg/L)	5.54	5.54	5.54	8.41	8.11	8.11	1.48	4.5	0.03390	*
pH	7.80	7.56	7.50	7.45	7.47	7.47	0.98	3.97059	0.04630	*
Salinity (ppt)	15.10	15.10	15.10	13.30	13.30	13.30	0.88	5	0.02535	*
Organic Matter (%)	3.71	3.86	3.80	9.93	9.81	9.90	2.61	3.85714	0.04954	*
Al (mg/L)	92.65	69.59	57.38	36.41	182.2	143.8	1.65	0.42857	0.51269	
As (mg/L)	0.10	0.03	0.00	0.00	0.08	0.02	0.10	0.19608	0.65791	
Ba (mg/L)	0.42	0.89	0.66	0.36	0.67	0.29	0.67	1.19048	0.27523	
Co (mg/L)	0.00	0.04	0.03	0.32	0.14	0.04	0.50	3.13726	0.07652	.
Cr (mg/L)	0.08	0.06	0.02	7.73	0.24	0.19	8.16	3.85714	0.04954	*
Cu (mg/L)	0.00	0.00	0.00	0.13	0.18	0.18	0.16	4.5	0.03390	*
Fe (mg/L)	94.99	82.40	58.98	228.0	206.5	157.3	2.50	3.85714	0.04954	*
Li (mg/L)	0.07	0.05	0.04	0.00	0.10	0.06	0.16	0.04762	0.82726	
Mn (mg/L)	0.92	1.17	0.64	5.13	2.31	0.67	2.97	1.19048	0.27523	
Ni (mg/L)	0.00	0.00	0.00	5.13	7.83	0.04	4.33	4.35484	0.03690	*
Pb (mg/L)	0.02	0.03	0.01	0.00	0.10	0.34	0.44	0.42857	0.51269	
Sn (mg/L)	0.00	0.00	0.00	0.00	0.00	0.09	0.03	1	0.31731	
V (mg/L)	0.16	0.17	0.13	0.74	0.43	0.26	1.43	3.857143	0.04954	*
Zn (mg/L)	1.01	0.63	0.57	0.62	0.56	0.42	0.72	2.333333	0.12663	

Table 1. Environmental variables.

Here we show the mean measures of environmental variables associated with the conserved (C1, C2, C3) and impacted (I1, I2, I3) sites used in this study as well as the ratio of impacted over conserved (I/C). These were tested using Krusakl-Wallis (df=1, H-statistic and p-value).

Environmental Variables	P1	P2	r2	Pr(>r)	Signif.
Temperature (C)	0.91786	-0.39690	0.9461	0.05000	*
Dissolved Oxygen (mg/L)	0.68884	0.72491	0.9012	0.06667	.
pH	-0.97386	0.22715	0.9368	0.06944	.
Salinity (ppt)	-0.68579	-0.72780	0.9060	0.10000	.
Organic Matter (%)	0.70743	0.70678	0.9077	0.03889	*
Al (mg/L)	0.09020	0.99592	0.1698	0.77083	
As (mg/L)	-0.87367	0.48651	0.4010	0.49722	
Ba (mg/L)	0.39804	-0.91737	0.7379	0.09583	.
Co (mg/L)	0.92968	0.36836	0.3930	0.40000	
Cr (mg/L)	0.75205	0.65911	0.2002	0.51667	
Cu (mg/L)	0.67743	0.73558	0.8690	0.05000	*
Fe (mg/L)	0.59300	0.80520	0.6798	0.10833	
Li (mg/L)	-0.84235	0.53893	0.0517	0.86806	
Mn (mg/L)	0.87229	0.48898	0.2456	0.52778	
Ni (mg/L)	0.77433	0.63278	0.3615	0.57500	
Pb (mg/L)	0.62015	0.78448	0.2883	0.44861	
Sn (mg/L)	0.52377	0.85186	0.2375	0.50000	
V (mg/L)	0.76228	0.64725	0.4700	0.37500	
Zn (mg/L)	-0.95954	0.28156	0.8378	0.16111	

Table 2. PCoA environmental variable vectors and significance.

Here we show the results of PCoA constrained ordination for OTUs for the environmental variables from the two sites.

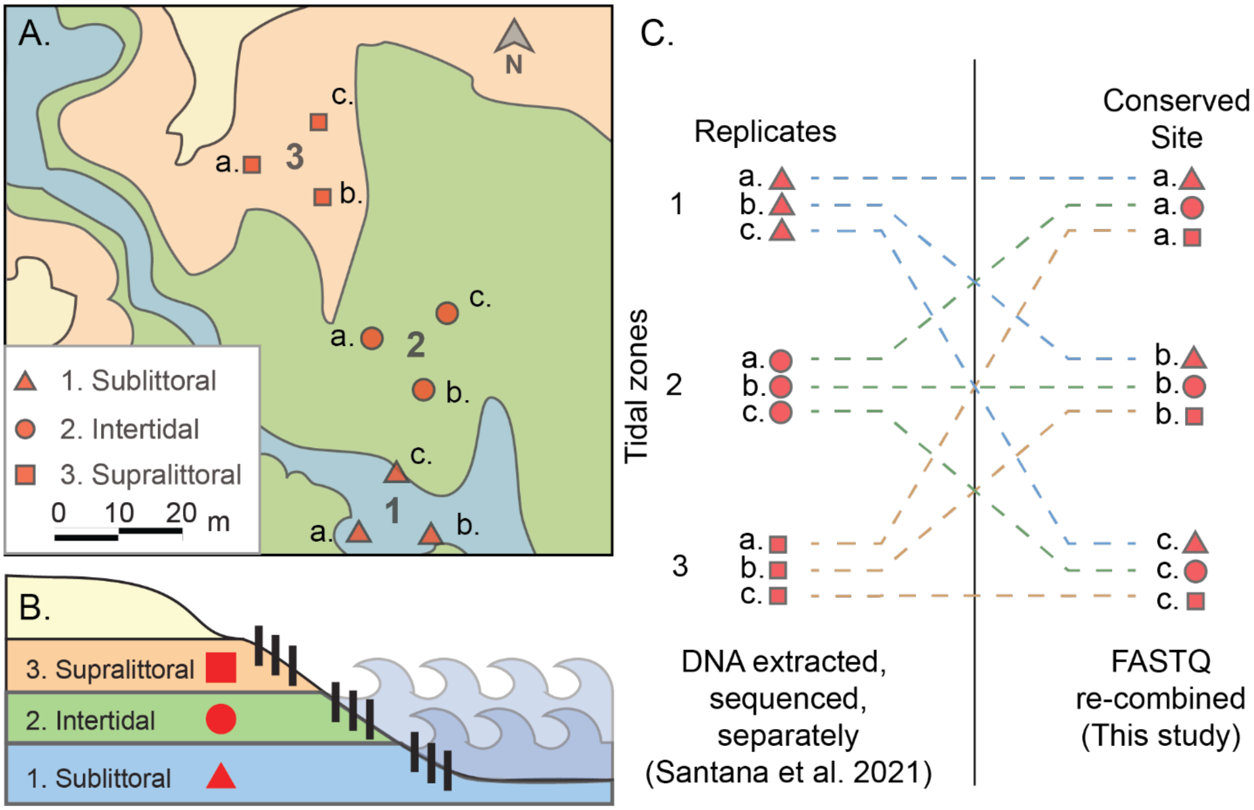

Figure 1. Sediment sampling strategy

Here we show the sampling schematic at the conserved site. Originally sampling was performed in triplicate at three different tidal zones (A, B) [1]. For this study we recombined samples such that each conserved site replicate had one sample from each tidal zone (C).

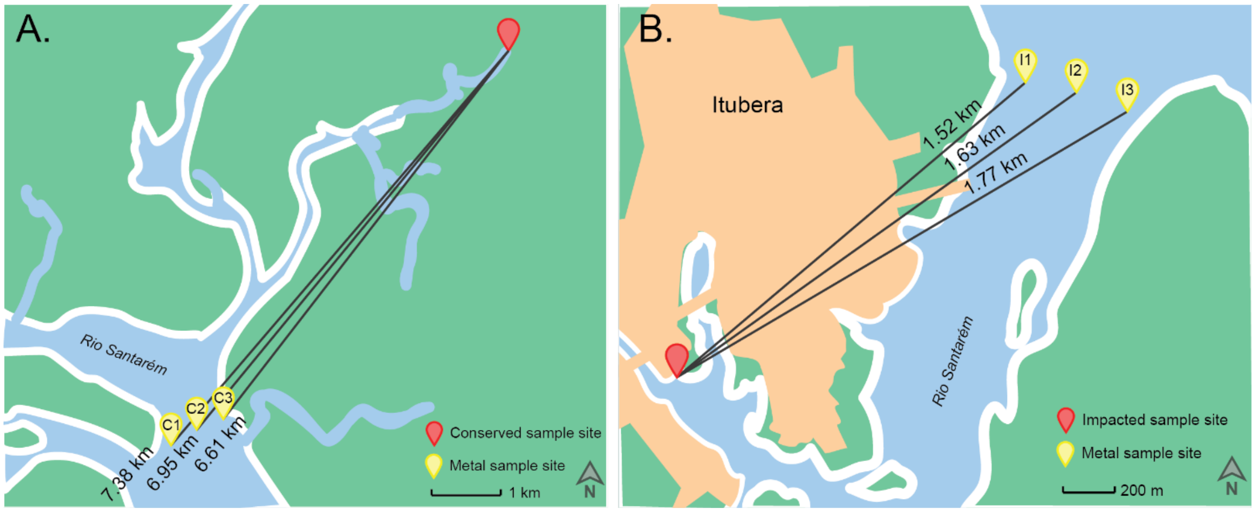

Figure 2. Schematics representing straight-line distances between sediment sampling sites and their respective heavy metal sampling sites.

Here we show the straight-line distances between the sediment sampling sites and their respective heavy metal sampling sites [2]. The average distance for the conserved set is 6.98 km (A) and 1.64 km for the Impacted site (B). In both cases the heavy metal sampling sites were selected based on being the closest sites downstream of the sediment sampling sites.

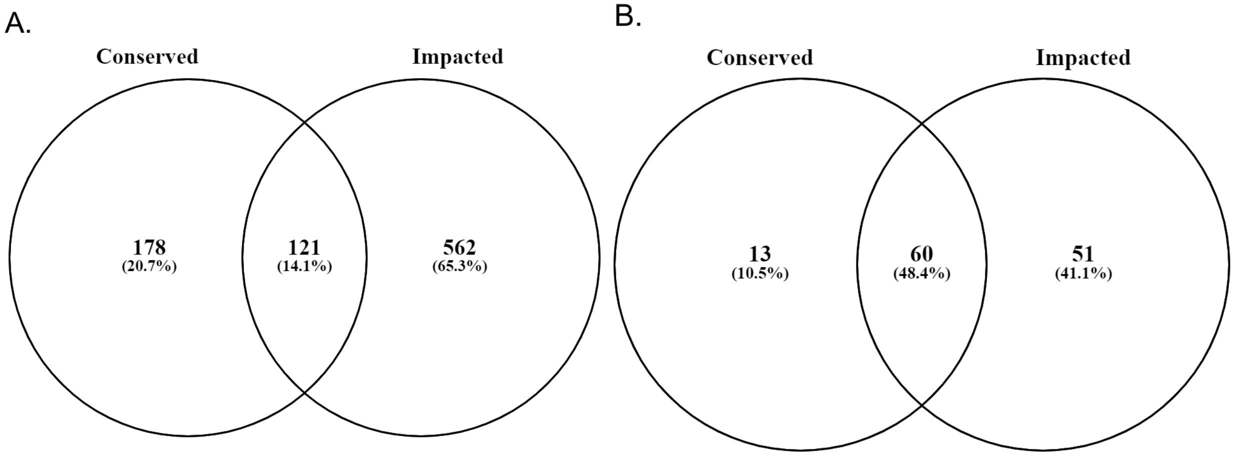

Figure 3. Taxa overlap between sites.
Here we show the overlap of taxa constrained to Species level and higher (A.) and Family level and higher (B.) Taxa were required to have a minimum of 20 unnormalized reads in at least one site to be included.

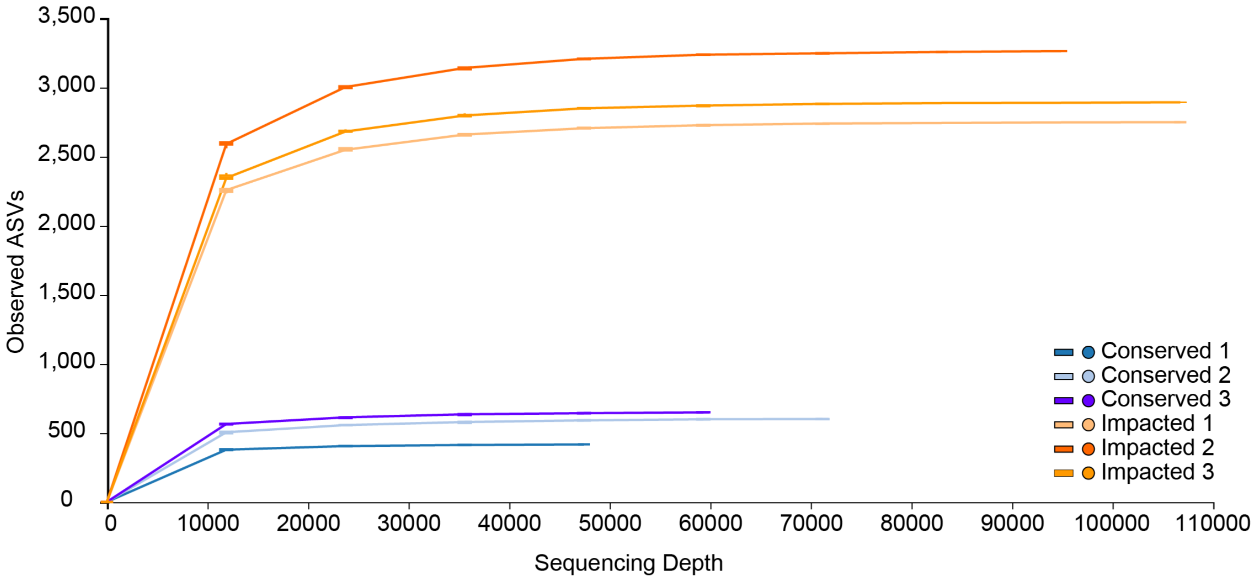

Figure 4. Alpha-rarefaction plot as calculated using QIIME2

Rarefaction curves for Impacted and Conserved sites. For rarefaction we selected Conserved 1 with 48,600 reads.

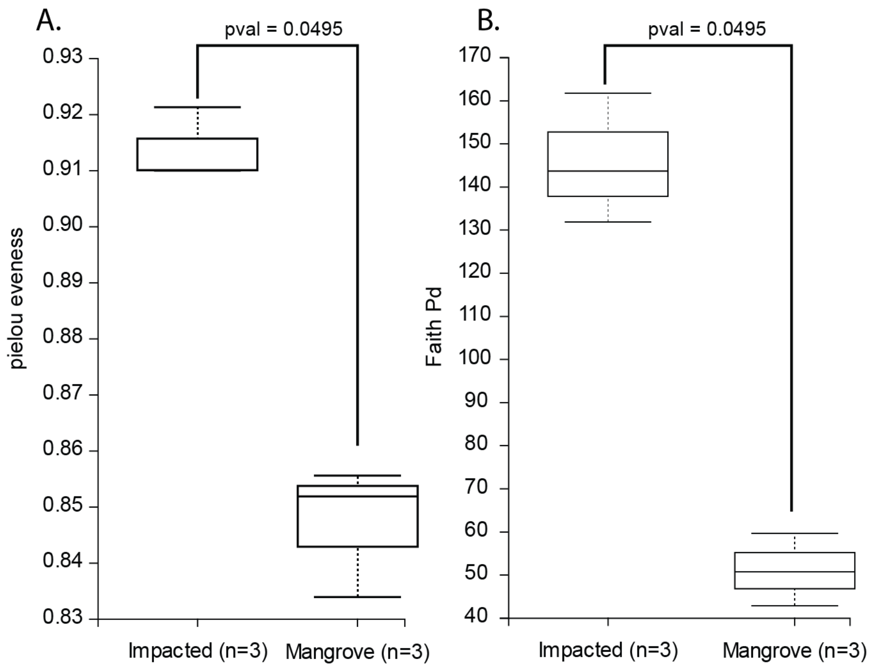

Figure 5. Additional alpha-diversity tests

In addition to Shannon's diversity, both Pielou (A) and Faith (B) were calculated using rarefied data and found to be significant using pairwise Kruskal-Wallis.

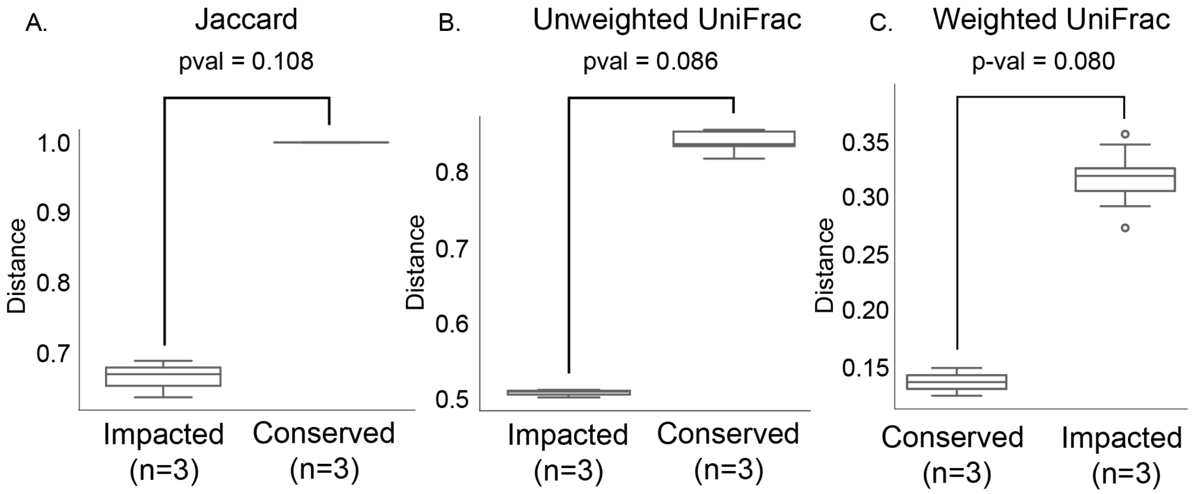

Figure 6. Additional beta-diversity tests

Distance measures for Jaccard (A), Unweighted UniFrac (B), and Weighted UniFrac (C). All p-values were calculated using the pairwise PERMANOVA test on rarefied data.

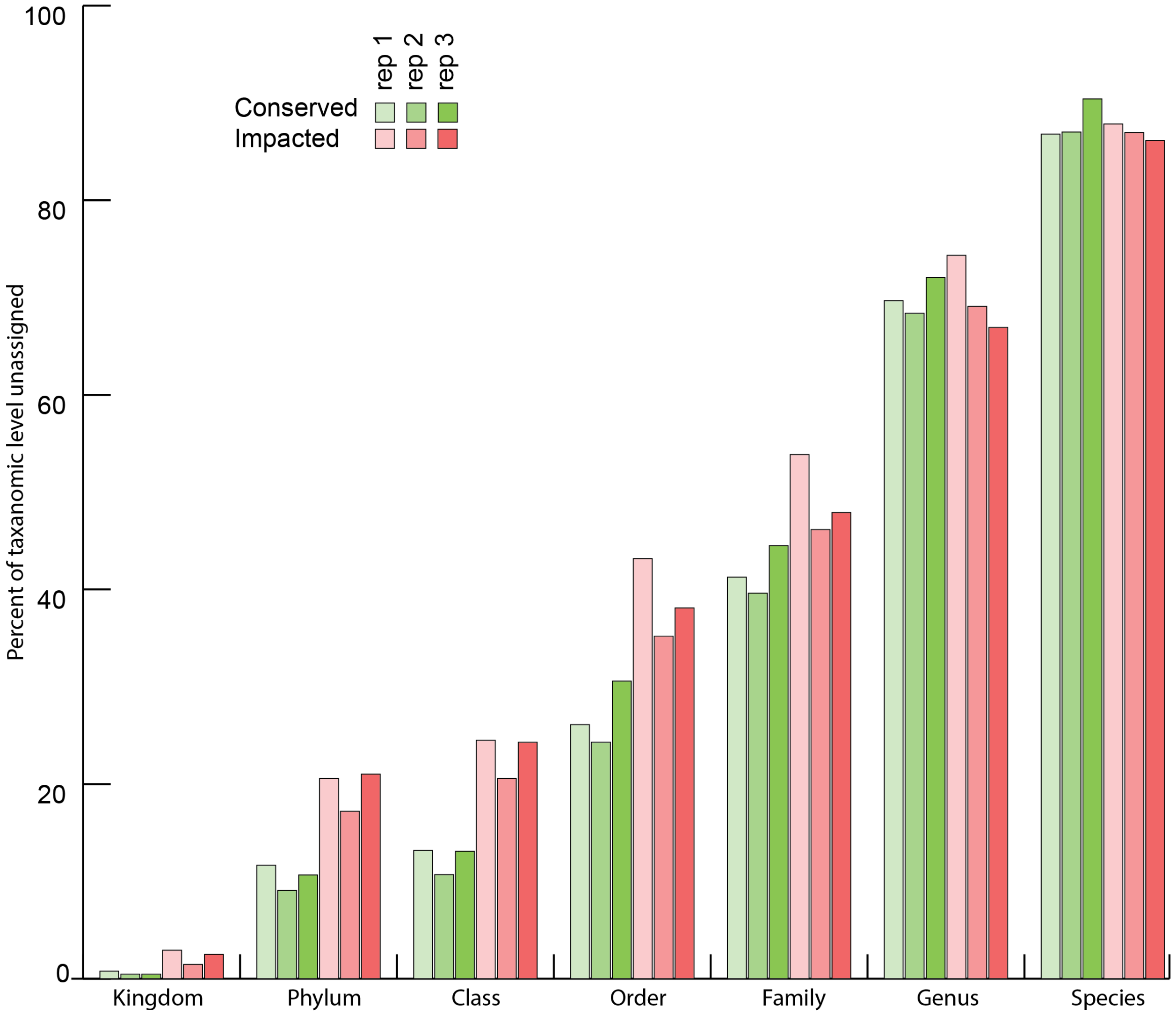

Figure 7. Percentage of reads belonging to unassigned ASVs at each taxonomic level.

Here we show the percent total of unnormalized abundance of reads for each ASV unassigned at that level. For example, a taxonomic assignment of ‘Bacteria’ would be counted as assigned at the Kingdom level but not for the Phylum level. No ASVs or OTUs were filtered from this calculation.

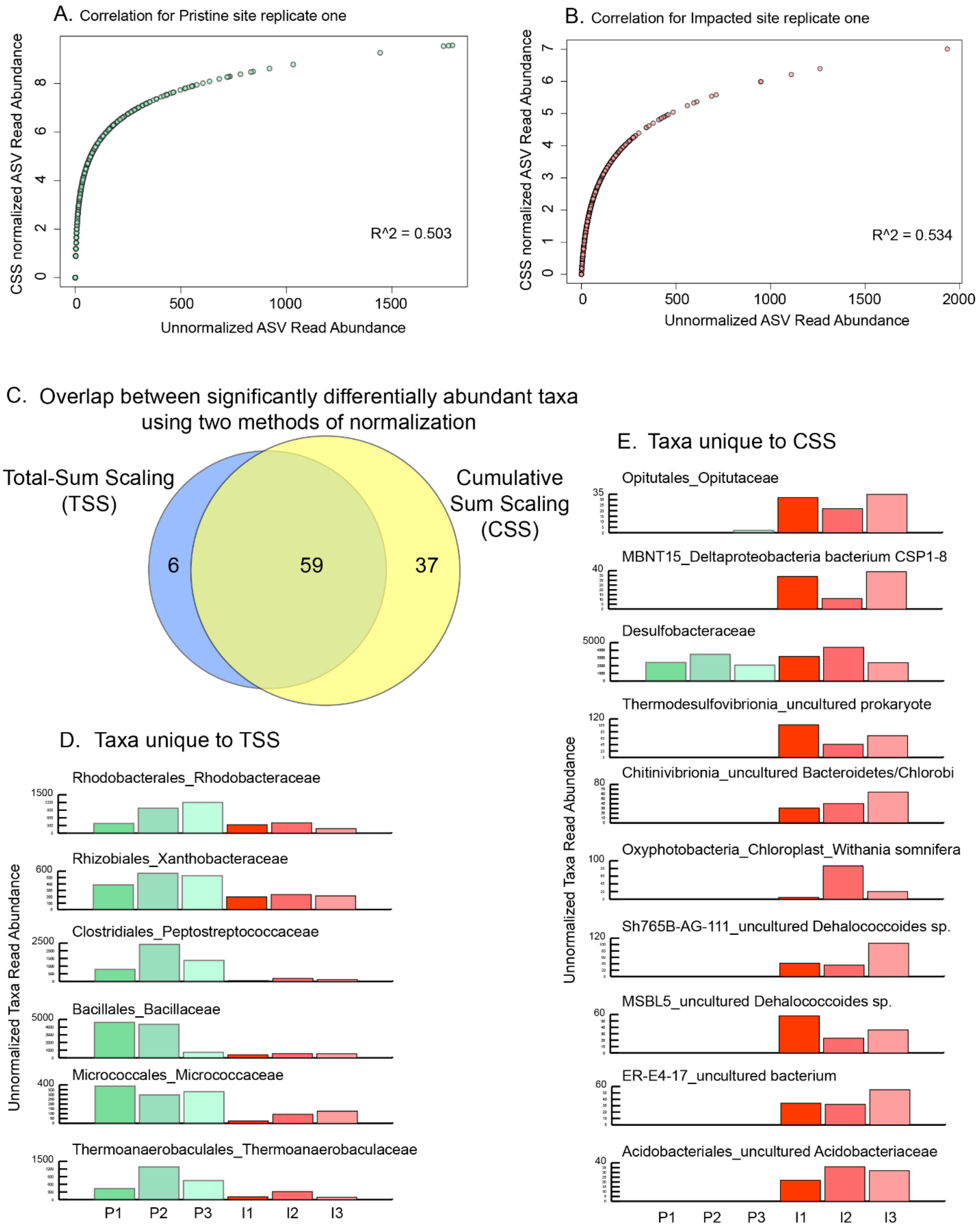

Figure 8. Analysis of Cumulative Sum Scaling on Differential Analysis.

Cumulative Sum Scaling (CSS) is a normalization method intended to correct for overamplification of sample specific sequences using an adaptive scaling term calculated around deviation from the reference sample [3].

Applied to our data we find that it significantly alters the distribution of feature scores, increasing weight of the lower abundance taxa relative to the abundance (A, B). The mean Pearson R^2 of unnormalized and CSS normalized ASV abundances: 0.503

Comparing performance of the methods in our differential analysis method (novembro.py) we find that they do have substantial overlap (~58%) (C). Normalization specific taxa do seem to have trends that describe them. The 6 TSS specific taxa have substantially higher unnormalized read abundance in the conserved site (minimum read count ~400) while also having one replicate closer in abundance to the impact replicates (D). As there are several dozen CSS specific taxa we randomly selected ten to show here. Of these we see that, with the exception of Desulfobacteraceae, these taxa are often not observed in the conserved samples and with low abundance in the impacted samples (maximum read count ~120) (E). Desulfobacteraceae, is an interesting case where the abundances in both samples are high. While CSS normalization increases this difference leading to it to be called significant, TSS decreases this difference leading to it being deemed not significant.

Because of this performance [4] we have decided to use TSS for normalization of our samples.

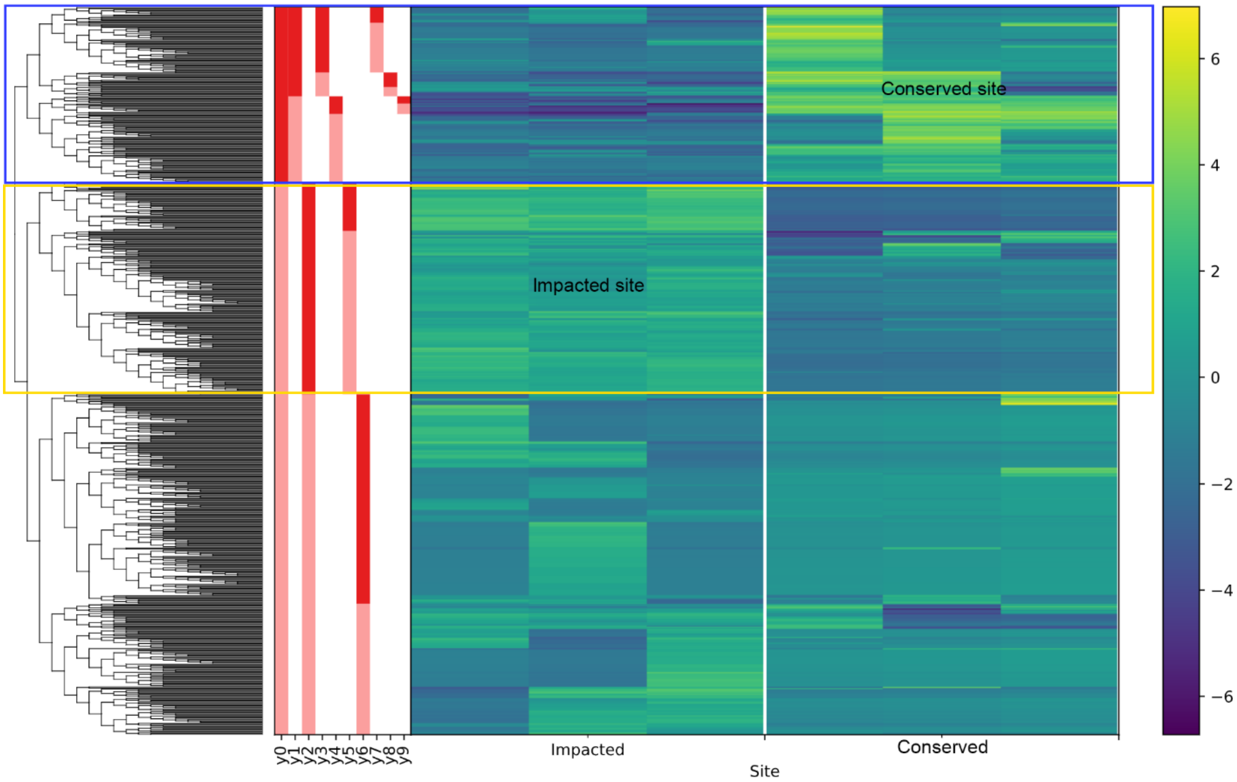

Figure 9. Differential subcommunity analysis using Gneiss. Gneiss is a hierarchical clustering approach that uses balance calculations to extend differential abundance analysis beyond species and instead identify niche specific subcommunities [5]. Here (Figure 3), we show the gneiss dendrogram, top ten balances, and heat plot of the our taxonomically assigned family's unnormalized read counts.

We find two highly ranked clusters that correspond to enrichment in the Conserved samples (blue box, numerator of y0, entirety of y1) and enrichment in the Impacted samples (orange box, numerator of y2, entirety of y5).

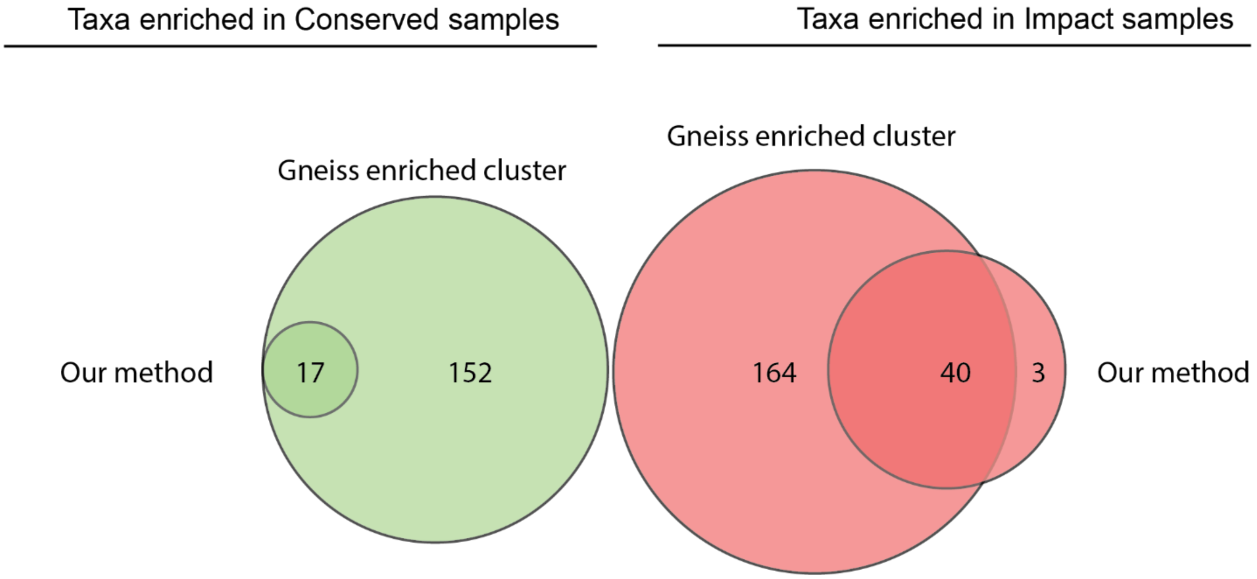

Figure 10. Overlap between Gneiss clusters and our differential abundance analysis method. We find 169 taxa are present in the Conserved site enriched cluster and 204 taxa are present in the Impacted site enriched cluster. Of these we find all (17) of our predicted Conserved enriched taxa are also present in the Conserved enriched cluster and all but 3 of our predicted Impact enriched taxa (43) are present in the Impact enriched cluster. Notably, those taxa (Bacteroidetes vadinHA17, Calditrichaceae, and Spirochaetaceae, Figure 4) which are absent from the Impacted enriched cluster are all taxa which also exhibit substantial abundances in the Conserved site, suggesting that they did not sort with this cluster due to that reason.

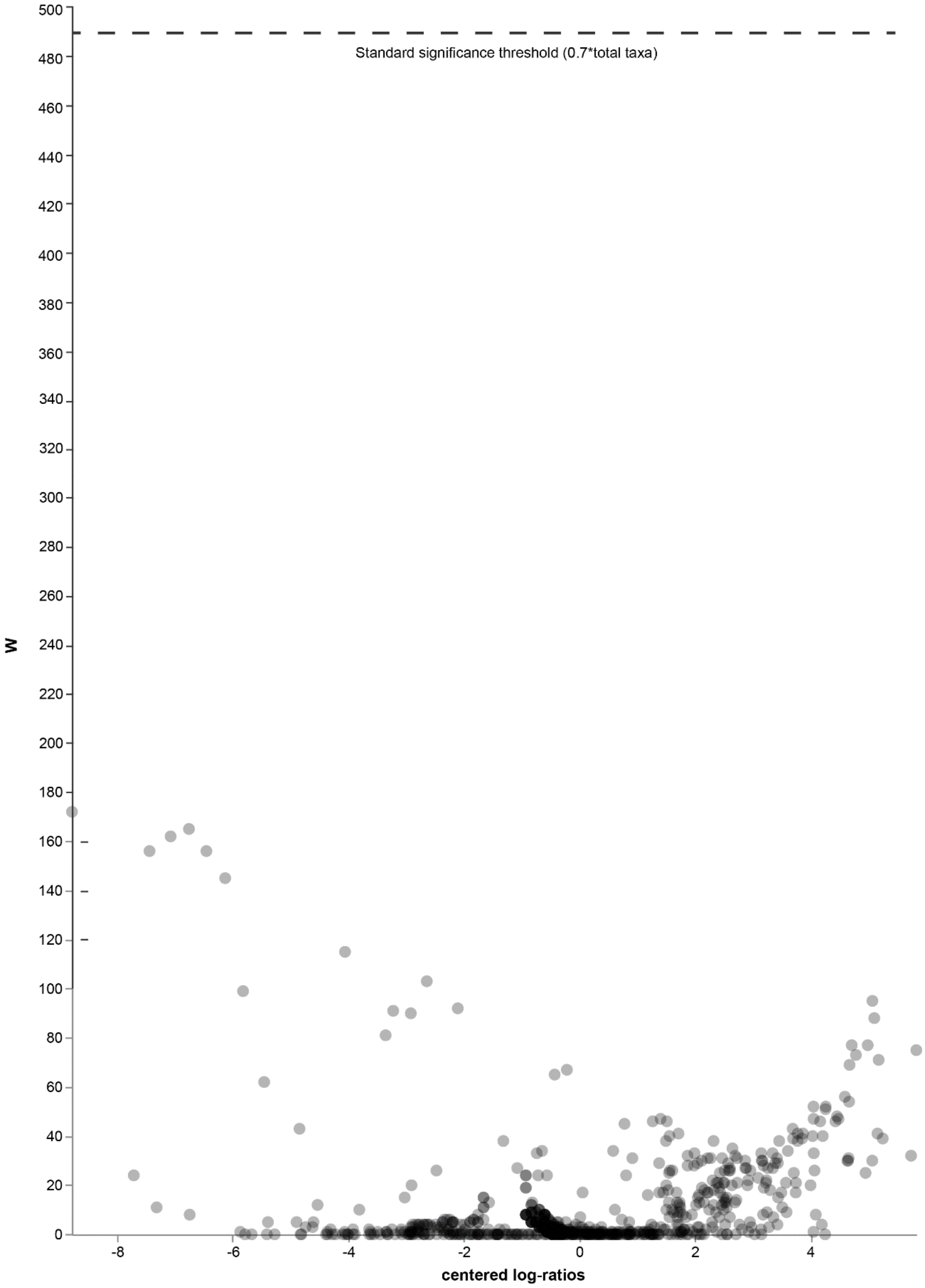

Figure 11. Results of ANCOMI applied to Family level constrained taxa unnormalized abundances.

Analysis of Composition of Microbiomes [6, 7] is a method of identifying taxa that have differential abundances between two sites. For normalization it uses a compositional approach instead of total-sum scaling (TSS) or distribution based models. While powerful, it assumes that the composition between sites is very similar with limited (<25%) differences in composition. As the taxa composition between or sites is high, ranging from 52% dissimilarity at the Family level to 86% dissimilarity at the species level, it was unclear if ANCOM would perform well given our data.

Unfortunately, this proved to be true. As both ANCOM (as implemented in QIIME2) and ANCOMII [4], resulted in no taxa being found to be significantly different, including those with obvious differences.

As one example, the Stappiaceae family of Alphaproteobacteria was observed in the following samples (unnormalized reads):

Conserved_rep1:		1744.0
Conserved_rep2:		2378.0
Conserved_rep3:		1781.0
Impacted_rep1:	0.0	
Impacted_rep2:	0.0	
Impacted_rep3:	31.0	

For this taxa, ANCOM only returns a W statistic of 24 for this taxa, far beneath the standard significance threshold (0.7*total taxa) of 492.

Given this performance we did not use ANCOM in our analysis.

Works cited

1. de Santana CO, Spealman P, Melo VMM, Gresham D, de Jesus TB, Chinalia FA. 2021. Effects of tidal influence on the structure and function of prokaryotic communities in the sediments of a pristine Brazilian mangrove. Biogeosciences 18:2259–2273. DOI: 10.5194/bg-18-2259-2021.

2. Pereira M. 2016. Espacialização geoquímica dos sedimentos do estuário do Rio Serinhaém, baixo sul da Bahia – Brasil / geochemical spacialization of the sediments of the Rio Serinhaém estuary, lower south of Bahia - Brazil. Programa de Pós-Graduação em Modelagem em Ciências da Terra e do Ambiente – PPGM Thesis. Universidade Estadual de Feira de Santana

3. Paulson, J., Stine, O., Bravo, H. et al. Differential abundance analysis for microbial marker-gene surveys. Nat Methods 10, 1200–1202 (2013). https://doi.org/10.1038/nmeth.2658

4. Weiss, S., Xu, Z.Z., Peddada, S. et al. Normalization and microbial differential abundance strategies depend upon data characteristics. Microbiome 5, 27 (2017). https://doi.org/10.1186/s40168-017-0237-y

5. Morton, James T et al. “Balance Trees Reveal Microbial Niche Differentiation.” mSystems vol. 2,1 e00162-16. 17 Jan. 2017, doi:10.1128/mSystems.00162-16

6. Mandal S, Van Treuren W, White RA, Eggesbø M, Knight R, Peddada SD. Analysis of composition of microbiomes: a novel method for studying microbial composition. Microb Ecol Health Dis. 2015 May 29;26:27663. doi: 10.3402/mehd.v26.27663. PMID: 26028277; PMCID: PMC4450248.

7. Kaul, Abhishek et al. “Analysis of Microbiome Data in the Presence of Excess Zeros.” Frontiers in microbiology vol. 8 2114. 7 Nov. 2017, doi:10.3389/fmicb.2017.02114
